# Supplementary material for: Physical development of infants born to patients with COVID-19 during pregnancy: 2 years of age
Source: PeerJ. 2024 Nov 12;12:e18481. doi: 10.7717/peerj.18481 (PMC11566510; doi:10.7717/peerj.18481)
Supplement: Supplemental Information 1 — Time intervals reported in months. [file peerj-12-18481-s001.docx]

**Supplemental Table 1.** Sample size of all reported growth chart measurements. Time intervals reported in months.

|  |  | Birth | 1-3 | 4-6 | 7-9 | 10-12 | 13-15 | 16-18 | 19-21 | 22-24 |
| --- | --- | --- | --- | --- | --- | --- | --- | --- | --- | --- |
| COVID-hospitalized  (N=39) | Weight | 39 | 32 | 28 | 24 | 15 | 14 | 15 | 9 | 5 |
|  | Length | 39 | 30 | 28 | 19 | 13 | 11 | 12 | 9 | 3 |
|  | HC | 34 | 28 | 28 | 17 | 16 | 10 | 9 | 6 | 2 |
| COVID-non-hospitalized  (N=719) | Weight | 719 | 542 | 490 | 392 | 312 | 205 | 197 | 142 | 99 |
|  | Length | 713 | 517 | 469 | 330 | 260 | 173 | 165 | 110 | 66 |
|  | HC | 632 | 499 | 456 | 340 | 282 | 186 | 148 | 67 | 24 |
| COVID+  (N=758) | Weight | 758 | 574 | 518 | 416 | 327 | 219 | 212 | 151 | 104 |
|  | Length | 752 | 547 | 497 | 349 | 273 | 184 | 177 | 119 | 69 |
|  | HC | 666 | 527 | 484 | 357 | 298 | 196 | 157 | 73 | 26 |
| Pandemic/COVID-  (N=9345) | Weight | 9248 | 6707 | 5807 | 4620 | 3871 | 3312 | 2662 | 1991 | 1302 |
|  | Length | 9221 | 6414 | 5543 | 4022 | 3303 | 2697 | 2140 | 1532 | 870 |
|  | HC | 8015 | 6055 | 5423 | 4030 | 3531 | 2459 | 2039 | 1004 | 281 |
| Pre-Pandemic  (N=3221) | Weight | 3086 | 2628 | 2496 | 2278 | 2096 | 2075 | 1887 | 1785 | 1516 |
|  | Length | 3079 | 2477 | 2331 | 1858 | 1721 | 1619 | 1481 | 1374 | 1015 |
|  | HC | 2468 | 2443 | 2334 | 1937 | 1884 | 1518 | 1428 | 892 | 326 |
