# Supplementary material for: Physical development of infants born to patients with COVID-19 during pregnancy: 2 years of age
Source: PeerJ. 2024 Nov 12;12:e18481. doi: 10.7717/peerj.18481 (PMC11566510; doi:10.7717/peerj.18481)
Supplement: Supplemental Information 2 — Wave designation based on infant’s DOB. *p<0.05, ** p < 0.01, *** p < 0.001 between infants born to COVID+ versus COVID- patients within each bolded column. Data reported as mean ± standard deviation or N (%). Annualized outcomes also report number of patients with outcome of interest (N). [file peerj-12-18481-s002.docx]

**Supplemental Table 2.** Infant outcomes by COVID-19 waves. Wave designation based on infant’s DOB. *p<0.05, ** p<0.01, *** p<0.001 between infants born to COVID+ versus COVID- patients within each bolded column. Data reported as mean ± standard deviation or N (%). Annualized outcomes also report number of patients with outcome of interest (N).

|  | **1^st^ Wave** | | **Alpha Wave** | | **Delta Wave** | | **Omicron Wave** | |
| --- | --- | --- | --- | --- | --- | --- | --- | --- |
|  | COVID+  (n=121) | COVID-  (n=822) | COVID+  (n=105) | COVID-  (n=1233) | COVID+  (n=73) | COVID-  (n=1867) | COVID+  (n=134) | COVID-  (n=325) |
| **Measurements at Birth** |  |  |  |  |  |  |  |  |
| Gestational Age (weeks) | 37.61±4.60 | 38.45±2.97 | 38.09±2.53 | 38.45±2.70 | 38.24±3.01 | 38.35±2.66 | 38.63±2.06 | 38.27±3.22 |
| LOS (days) | 2.37±2.52 | 2.10±1.52 | 2.33±1.47 | 2.34±1.92 | 2.42±1.56 | 2.39±1.73 | 2.54±2.28 | 2.30±1.84 |
| Weight (g) | 3094.69±724.09 | 3109.61±704 | 3064.76±658 | 3138.39±654 | 3134.60±739 | 3084.79±691 | 3104.25±618 | 3134.60±739 |
| Length (cm) | 48.97±6.24 | 48.66±3.81 | 47.67±3.76 | 48.29±7.68 | 47.52±4.70 | 47.61±3.67 | 47.39±3.74 | 47.79±3.51 |
| Head Circumf(cm) | 33.13±2.70 | 33.53±2.39 | 33.77±2.32 | 33.65±2.46 | 33.71±2.62 | 33.70±2.29 | 33.59±2.08 | 33.71±2.16 |
| **Outcomes at Birth** |  |  |  |  |  |  |  |  |
| Critical Care | 9 (7.44%) | 40 (4.87%) | 5 (4.76%) | 63 (5.11%) | 5(6.85%) | 65(3.48%) | 4 (2.99%) | 10 (3.08%) |
| Preterm Birth | 26 (21.49%) | 103 (12.53%)* | 18 (17.14%) | 150 (12.17%) | 10 (13.70%) | 276 (14.78%) | 11 (8.21%) | 43 (13.23%) |
| Birth weight <2500 g | 20 (16.53%) | 101(12.29%) | 12 (12.38%) | 142 (11.51%) | 12 (16.44%) | 253 (13.54%) | 15 (11.19%) | 44 (13.54%) |
| Stillbirth | 0 (0%) | 7 (0.85%) | 0 (0%) | 10 (0.81) | 0 (0%) | 14 (0.75%) | 0 (0%) | 7 (2.15%) |
| Cesarean Delivery | 51 (42.15%) | 286 (34.79%) | 40 (38.09%) | 445 (36.09%) | 32 (43.83%) | 665 (35.61%) | 50 (37.31%) | 114 (35.07%) |
| **Annualized Outcomes** |  |  |  |  |  |  |  |  |
| ED Visit | 1.19±1.95 (58) | 1.01±2.27 (364) | 1.57±2.11 (55) | 1.47±2.28 (589) | 2.43±3.55 (41) | 2.19±3.80 (880) | 2.58±3.83 (64) | 2.03±3.39 (127) |
| Hospitalization | 0.88±0.81 (121) | 0.80±1.12 (812) | 1.19±0.90 (105) | 1.14±0.96 (1233) | 1.76±1.63 (73) | 1.69±1.58 (1850) | 2.10±1.69 (134) | 1.86±1.40 (318) |
| LOS (days) | 4.18±13.48 | 3.13±9.25 | 3.93±8.11 | 3.42±8.72 | 2.95±5.65 | 3.40±8.40 | 3.39±7.99 | 2.90±5.98 |
| Outpatient Visits | 16.32±15.66 (120) | 13.08±16. (811) | 15.58±15.(104) | 15.62±15 (1217) | 20.21±17 (72) | 21.04±21 (1844) | 23.47±17 (132) | 25.32±25. (313) |
| Symptom/disease |  |  |  |  |  |  |  |  |
| GI Symptoms | 0.19±0.65 (15) | 0.22±0.76 (127) | 0.29±0.78 (19) | 0.24±0.73 (168) | 0.37±1.06 (10) | 0.36±1.28 (242) | 0.25±0.79 (14) | 0.34±1.30 (33) |
| Fever | 0.19±0.48 (25) | 0.18±0.46 (153) | 0.24±0.49 (22) | 0.22±0.62 (189) | 0.22±0.58 (10) | 0.30±0.98 (265) | 0.34±1.06 (17) | 0.39±1.09 (47) |
| Jaundice | 0.23±0.56 (24) | 0.19±0.59 (123) | 0.49±1.07 (26) | 0.41±1.27 (215) | 0.73±2.31 (14) | 0.69±2.08 (333) | 0.64±1.67 (22) | 0.97±2.51 (66) |
| Dehydration | 0.02±0.14 (9) | 0.02±0.17 (12) | 0.03±0.17 (0) | 0.02±0.19 (0) | 0±0 (0) | 0.03±0.27*** (0) | 0.07±0.38 (0) | 0.04±0.43 (0) |
| Bronchiolitis | 0.07±0.35 (8) | 0.05±0.26 (39) | 0.14±0.66 (9) | 0.11±0.46 (89) | 0.22±0.90 (6) | 0.24±1.02 (160) | 0.37±1.47 (10) | 0.19±0.50 (21) |
| Respiratory symptoms | 0.47±1.08 (36) | 0.36±1.00 (188) | 0.41±1.10 (22) | 0.45±1.11 (301) | 0.81±1.62 (19) | 0.66±1.92 (434) | 0.71±1.54 (34) | 0.89±2.10* (82) |
| Viral Infection |  |  |  |  |  |  |  |  |
| SARS-CoV-2 | 0.02±0.09 (9) | 0.02±0.09 (45) | 0.03±0.13 (7) | 0.04±0.16 (95) | 0.08±0.31 (6) | 0.09±0.30 (184) | 0.14±0.39 (17) | 0.09±0.30 (29) |
| Influenza | 0.05±0.14 (14) | 0.04±0.16 (64) | 0.09±0.26 (13) | 0.07±0.26 (126) | 0.14±0.44 (8) | 0.18±0.58 (251) | 0.31±0.82 (24) | 0.29±0.89 (50) |
| RSV | 0.02±0.15 (3) | 0.02±0.11 (20) | 0.03±0.22 (2) | 0.03±0.20 (31) | 0.12±0.58 (4) | 0.07±0.43 (58) | 0.06±0.42 (2) | 0.11±0.56 (14) |
| Pneumovirus | 0±0 (0) | 0±0 (0) | 0±0 (0) | 0.0012±0.025* (3) | 0±0 (0) | 0.0024±0.045* (6) | 0±0 (0) | 0.009±0.096* (3) |
| Adenovirus | 0±0 (0) | 0.01±0.09*** (4) | 0±0 (0) | 0.004±0.04*** (10) | 0.01±0.12 (1) | 0.004±0.08 (8) | 0.007±0.09 (1) | 0.009±0.096 (3) |
| MRSA | 0.017±0.11 (3) | 0.004±0.075 (4) | 0±0 (0) | 0.0048±0.13 (4) | 0±0 (0) | 0.0048±0.11* (5) | 0±0 (0) | 0.012±0.22 (1) |
