# Supplementary figures and images for: Physical development of infants born to patients with COVID-19 during pregnancy: 2 years of age
Source: PeerJ. 2024 Nov 12;12:e18481. doi: 10.7717/peerj.18481 (PMC11566510; doi:10.7717/peerj.18481)

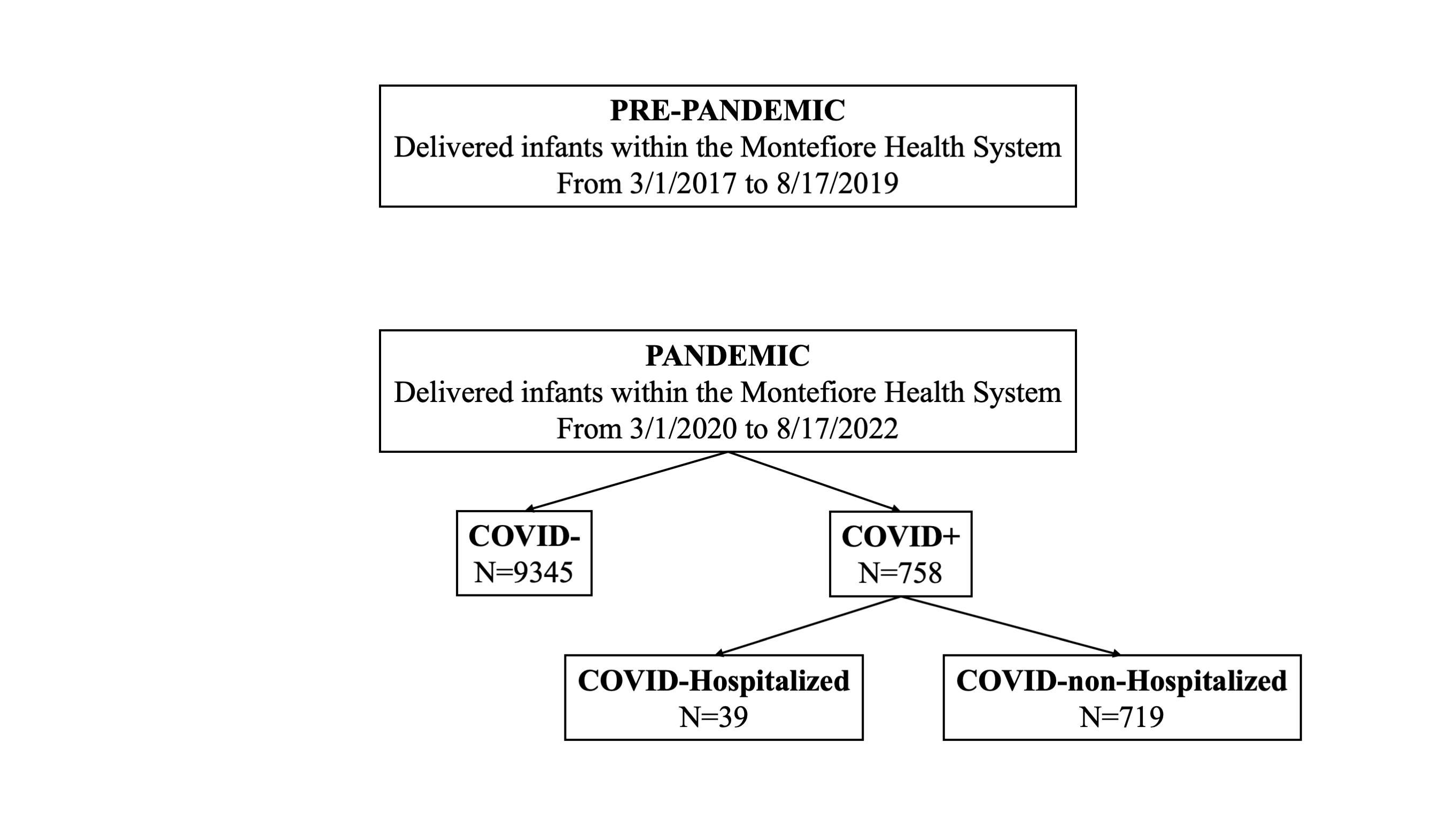

Supplement: Supplemental Information 3 — The COVID+ and hospitalization status was that of the birthing persons’. [file peerj-12-18481-s003.tiff]
